# Evaluation of bempedoic acid in elderly patients: real-world evidence from the REALIST study

**DOI:** 10.3389/fragi.2026.1675989

**Published:** 2026-03-11

**Authors:** Saverio Muscoli, Emanuele Di Marco, Fiorella Puttini, Sara Sposini Ghezzi, Mihaela Ifrim, Mobina Amtaeh, Emanuele Maria Renga, Caterina Cappello, Giulio Barone, Gaetano Chiricolo, Giuseppe Massimo Sangiorgi, Andrea Natale, David Della-Morte

**Affiliations:** 1 Division of Cardiology, Policlinico Tor Vergata, Rome, Italy; 2 Division of Geriatrics and Clinical Nutrition, Department of Medical Sciences, University Hospital Fondazione Policlinico Tor Vergata, Rome, Italy; 3 Department of Biomedicine and Prevention, University Tor Vergata, Rome, Italy; 4 Case Western Reserve University School of Medicine, Cleveland, OH, United States; 5 Department of Neurology, Evelyn F. McKnight Brain Institute, Miller School of Medicine, University of Miami, Miami, FL, United States

**Keywords:** bempedoic acid (BA), cardiovascular risk management, elderly patient, high cardiovascular risk, real-world data (RWD), statin intolerance

## Abstract

Statin intolerance and PCSK9 inhibitor reimbursement restrictions limit lipid-lowering options for elderly patients at high cardiovascular (CV) risk. Bempedoic acid (BA), an ATP-citrate lyase inhibitor, is a promising alternative. This study analyzed 54 patients over 81 years, selected from 2,564 individuals with medium to very high CV risk, who received BA for 52 weeks. Lipid profiles, renal and hepatic function, and adverse events were assessed. BA significantly reduced Low-Density Lipoprotein Cholesterol (LDL-C), triglycerides, and total cholesterol while maintaining stable HDL-C levels. Renal and hepatic function remained unchanged, with increased uric acid as the only notable adverse event. No treatment discontinuations occurred. During follow-up, five patients underwent coronary angiography, two developed atrial fibrillation, and two were hospitalized for heart failure. BA demonstrated efficacy and tolerability in this high-risk, elderly population. It represents a viable lipid-lowering option for statin-intolerant patients or those ineligible for PCSK9 inhibitors. Further studies are needed to confirm long-term cardiovascular benefits.

## Highlights

- Bempedoic acid significantly reduced LDL-C, triglycerides, and total cholesterol levels in elderly patients aged 81 years or older who were at high cardiovascular risk.
- The study population, largely statin-intolerant or ineligible for PCSK9 inhibitors, demonstrated high treatment adherence and no discontinuations.
- Renal and hepatic functions remained stable over 52 weeks; the only clinically relevant adverse effect was increased uric acid, which was manageable without treatment withdrawal.
- Real-world evidence supports bempedoic acid as a safe and effective lipid-lowering option in patients with limited alternatives due to age or intolerance.
- The findings address a critical therapeutic gap in lipid management among elderly populations, which are often excluded from randomized clinical trials.

## Introduction

Cardiovascular diseases (CVDs) are a leading cause of morbidity and mortality worldwide, and dyslipidemia represents one of the major modifiable risk factors in their pathogenesis (Saeed et al., 2018; Cimmino et al., 2023).

Statins remain the cornerstone of lipid-lowering therapy due to their proven efficacy in lowering low-density lipoprotein cholesterol (LDL-C) levels and reducing cardiovascular risk (Laufs et al., 2019).

However, a substantial proportion of patients, particularly the elderly, experience intolerance (SI) to statins, primarily due to muscle-related adverse effects such as myalgia, liver dysfunction, and cognitive impairment (Banach et al., 2015).

SI often results in poor adherence or discontinuation of therapy, preventing many older patients from achieving guideline-recommended LDL-C targets (Mancini et al., 2016; Banach and Mikhailidis, 2018).

Alternative lipid-lowering strategies have been developed to address this challenge (Muscoli et al., 2022).

Ezetimibe, an inhibitor of intestinal cholesterol absorption, is commonly used as a second-line therapy but may be insufficient as monotherapy for high-risk patients (Giugliano et al., 2018; Perez-Calvo et al., 2005).

Novel drugs targeting proprotein convertase subtilisin/kexin type 9 (PCSK9), such as evolocumab, alirocumab and inclisiran, provide substantial LDL-C reduction with a favourable safety profile; however, their high cost and restricted reimbursement policies, particularly in individuals over 80 years of age in many countries, limit their widespread use in the elderly population (Gargiulo et al., 2024a; Filardi et al., 2024; Gargiulo et al., 2024b).

Reimbursement criteria (Italian Medicines Agency [AIFA], GU 231–03.10.2022) include patients at very high cardiovascular (CV) risk with LDL-C of ≥70 mg/dL on statin ± ezetimibe less than 81 years old.

Consequently, there remains an unmet clinical need for effective and well-tolerated lipid-lowering therapies in elderly patients, particularly those who are statin-intolerant or unable to access PCSK9 inhibitors. Bempedoic acid (BA) has recently emerged as a promising alternative for lipid management (Laufs et al., 2019).

As an adenosine triphosphate-citrate lyase (ACLY) inhibitor, BA reduces hepatic cholesterol synthesis upstream of HMG-CoA reductase, the enzyme targeted by statins (Ferri et al., 2024).

Unlike statins, BA is a prodrug that requires activation in the liver but is inactive in skeletal muscle, thereby reducing the risk of muscle-related adverse effects (Ferri et al., 2024).

Clinical studies have demonstrated that BA lowers LDL-C levels by approximately 18%–28% when administered as monotherapy or in combination with ezetimibe (De Filippo et al., 2023).

In addition, BA has been shown to significantly reduce levels of high-sensitivity C-reactive protein (hs-CRP), an important marker of systemic inflammation that is particularly important in older patients with chronic, low-grade inflammation and increased CV risk (Pinkosky et al., 2016).

BA, especially in elderly patients, may allow the LDL-C target values recommended by the guidelines to be achieved with a lower statin dose and thus reduce the likelihood of developing statin-associated muscle symptoms (SAMS (Nicholls et al., 2021)).

Despite these promising findings, data on the efficacy and safety of BA in elderly patients, particularly those over 81 years, remain limited (Ray et al., 2023).

Given the high prevalence of SI in this population and the regulatory restrictions on PCSK9 inhibitors, BA may represent a valuable therapeutic alternative.

However, real-world evidence assessing its lipid-lowering efficacy, safety profile, and long-term cardiovascular benefits in this specific demographic is still lacking.

This study evaluates the safety and efficacy of BA in elderly patients at high cardiovascular risk, focusing on those over 81 years who cannot tolerate high-dose statins or are ineligible for PCSK9 inhibitors. By assessing lipid-lowering efficacy, safety outcomes, and cardiovascular risk markers, it aims to determine whether BA can bridge the existing therapeutic gap in lipid management for this high-risk population.

## Materials and methods

This observational study was designed and reported in accordance with the STROBE (Strengthening the Reporting of Observational Studies in Epidemiology) guidelines.

### Study design and setting

This retrospective observational cohort study was conducted using data from the REALIST registry (REgistro cardiology dISlipidemia Tor Vergata), a single-center registry at the Cardiology Department of Policlinico Tor Vergata, Rome, Italy. The registry captures detailed clinical, demographic, and treatment information of patients undergoing lipid-lowering therapy.

### Participants

Between September 2021 and December 2023, a total of 2,564 patients with medium to very high cardiovascular (CV) risk were evaluated. From this population, 182 patients treated with bempedoic acid (BA) were identified. Inclusion criteria for this analysis comprised age >81 years, high or very high CV risk as defined by ESC/EAS guidelines, and ongoing BA therapy for at least 52 weeks. Patients eligible for PCSK9 inhibitors under Italian Medicines Agency (AIFA) reimbursement criteria were excluded. A final cohort of 54 elderly patients met the inclusion criteria.

Patients included in the REALIST registry were consecutively enrolled during outpatient screening visits following hospital admission, as part of routine cardiovascular follow-up. The registry captures real-world data from an ambulatory cardiology setting, minimizing selection bias.

### Variables and data sources

Data were extracted from electronic health records and the REALIST registry. Baseline and follow-up variables included demographic data (age, sex, BMI), comorbidities (diabetes, hypertension, coronary artery disease, peripheral artery disease, cerebrovascular disease), and concurrent medications. Laboratory parameters comprised LDL-C, HDL-C, total cholesterol, triglycerides, creatinine, estimated glomerular filtration rate (eGFR), alanine aminotransferase (ALT), aspartate aminotransferase (AST), and uric acid levels. Cardiovascular outcomes and adverse events were also recorded.

### Outcome measures

Primary outcomes included changes in lipid parameters (LDL-C, total cholesterol, triglycerides, HDL-C) from baseline to 52 weeks. Secondary outcomes encompassed renal and hepatic function stability, adverse events, and cardiovascular events including hospitalization for heart failure, atrial fibrillation, and coronary angiography.

### Bias

Potential sources of bias include selection bias due to the single-center nature of the registry and reporting bias from reliance on recorded data. To mitigate these, only patients with complete 52-week follow-up and laboratory evaluations were included.

### Study size

The sample size of 54 patients over 81 years was determined by the availability of patients meeting inclusion criteria within the registry during the study period. No formal sample size calculation was conducted.

### Quantitative variables

Continuous variables were summarised as mean ± standard deviation or median and interquartile range, as appropriate. Categorical variables were expressed as counts and percentages.

### Ethical considerations

The study protocol adhered to the principles outlined in the Declaration of Helsinki. Written informed consent was obtained from all participants, and the local ethics committee of Policlinico Tor Vergata approved the study.

### Statistical analysis

Descriptive statistics summarise the data. Continuous variables are expressed as mean ± standard deviation or median and interquartile range, as appropriate. Paired continuous variables were compared using the paired Student’s t-test or Wilcoxon signed-rank test, as appropriate. Categorical variables are presented as frequencies and percentages and were compared using the Chi-squared test. Differences were considered statistically significant at p < 0.05. Statistical analyses were performed using the Statistical Package for the Social Sciences (SPSS), version 26 (IBM Corp., Armonk, NY, USA).

## Results

### Baseline characteristics

Table 1 summarizes the baseline characteristics of the study population. In the subgroup of patients over 81 years, 30% were female, with a mean body mass index (BMI) of 25 ± 6 kg/m^2^. Comorbidities were highly prevalent: 51 out of 54 patients (94%) had coronary artery disease, 8 (14.8%) had peripheral artery disease, and 3 (5.5%) had cerebrovascular atherosclerotic disease. Additionally, 39 patients (72.2%) had diabetes, and 49 (90.7%) had hypertension.

**TABLE 1:** Baseline demographic and clinical characteristics of patients over 81 years treated with BA. Data are expressed as mean ± standard deviation (SD) for continuous variables and as percentages for categorical variables.

Characteristic	Baseline values
Age (years)	>81 (inclusion criterion)
Female (%)	30%
BMI (kg/m^2^)	25 ± 6
Coronary artery disease (%)	94%
Peripheral artery disease (%)	14.8%
Cerebrovascular disease (%)	5.5%
Diabetes (%)	72.2%
Hypertension (%)	90.7%
Statin use (%)	72.2%
Ezetimibe use (%)	27.8%
LDL-C (mg/dL) ±SD	88 ± 8
HDL-C (mg/dL) ±SD	41 ± 5
Triglycerides (mg/dL) ±SD	139 ± 21
Total cholesterol (mg/dL) ±SD	158 ± 17
Creatinine (mg/dL) ±SD	1.2 ± 0.8
GFR (mL/min/1.73m^2^) ±SD	58 ± 15
ALT (U/L) ±SD	31 ± 8
AST (U/L) ±SD	33 ± 6
Uric acid (mg/dL) ± SD	5.9 ± 2.7

BMI, body mass index; LDL-C, low-density lipoprotein cholesterol; HDL-C, high-density lipoprotein cholesterol; GFR, glomerular filtration rate; ALT, alanine aminotransferase; AST, aspartate aminotransferase.

Regarding lipid-lowering therapy, 39 out of 54 patients (72.2%) were on statins, while 15 (27.8%) received ezetimibe monotherapy due to statin intolerance. Overall, 49 patients (90%) were treated with ezetimibe alone or combined with statins. The recorded biochemical parameters for this subgroup were as follows: LDL-C, 88 ± 8 mg/dL; HDL cholesterol, 41 ± 5 mg/dL; triglycerides, 139 ± 21 mg/dL; total cholesterol, 158 ± 17 mg/dL; Creatinine 1.2 ± 0.8 mg/dL; glomerular filtration rate (GFR), 58 ± 15 mL/min/1.73 m^2^; alanine aminotransferase (ALT), 31 ± 8 U/L; aspartate aminotransferase (AST), 33 ± 6 U/L; and uric acid, 5.9 ± 2.7 mg/dL.

### 52-Week outcomes

**FIGURE 1:**
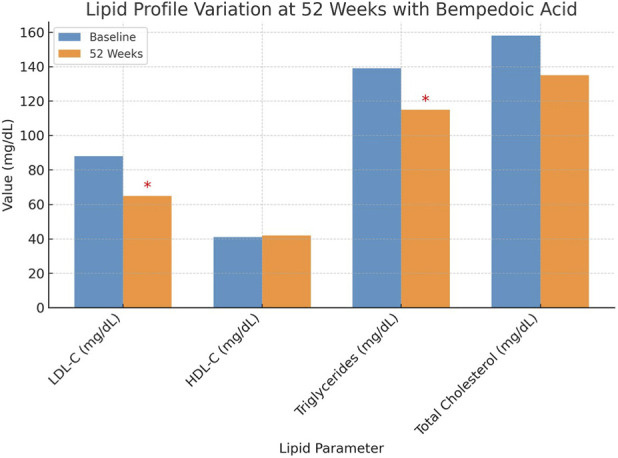
Lipid Profile Variation at 52 Weeks with Bempedoic Acid Changes in lipid parameters from baseline to 52 weeks in patients treated with BA. A significant reduction in LDL-C, triglycerides, and total cholesterol was observed (marked with * for p < 0.05), while HDL-C levels remained stable.

**FIGURE 2:**
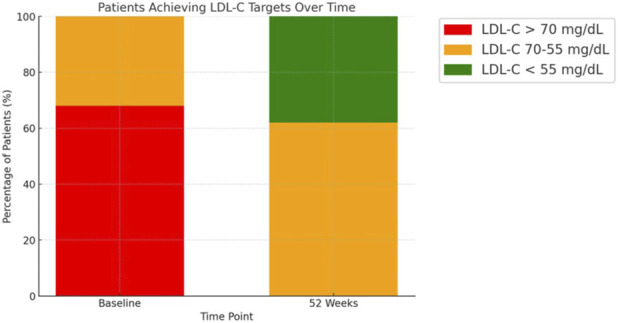
Patients Achieving LDL-C Targets Over Time. Distribution of patients based on LDL-C thresholds at baseline and after 52 weeks of treatment with bempedoic acid. At baseline, the majority of patients had LDL-C levels >70 mg/dL (red) or between 55 and 70 mg/dL (orange). After 52 weeks, a substantial proportion of patients achieved LDL-C levels <70 mg/dL (blue) or <55 mg/dL (green), highlighting the treatment's efficacy in achieving lipid-lowering targets.

After 52 weeks of treatment with BA, patients exhibited significant improvements in their lipid profiles, Figure 1. The mean LDL-C levels decreased from 88 ± 8 mg/dL to 63 ± 9 mg/dL (p < 0.0001). Triglyceride levels also reduced from 139 ± 21 mg/dL to 111 ± 12 mg/dL (p < 0.0001), while total cholesterol significantly declined from 158 ± 17 mg/dL to 148 ± 9 mg/dL (p = 0.0028). In contrast, HDL-C levels remained stable, showing no significant change (HDL 42 ± 13 in HDL 42 ± 3). The impact of BA treatment on LDL-C target achievement is illustrated in Figure 2. At baseline, the majority of patients had LDL-C levels above the recommended thresholds, with a substantial proportion exceeding 70 mg/dL. After 52 weeks, a significant shift was observed, with most patients achieving LDL-C levels below 70 mg/dL, and a considerable subset reaching levels below 55 mg/dL. Regarding safety, renal function and liver enzyme levels remained stable throughout the study period. Serum creatinine levels showed no significant variation (1.2 ± 0.8 mg/dL vs. 1.2 ± 0.6 mg/dL, p = 0.149), and the estimated glomerular filtration rate (GFR) remained unchanged (58 ± 15 vs 56 ± 12 mL/min/1.73 m^2^, p = 0.870). Similarly, ALT (p = 0.675) and AST levels (p = 0.254) did not significantly change. However, a significant increase in uric acid levels was observed, rising from 5.9 ± 2.7 mg/dL to 6.8 ± 2.8 mg/dL (p = 0.0172), which required pharmacological management in some cases but did not lead to treatment discontinuation.

During the follow-up period, five patients underwent coronary angiography, two developed atrial fibrillation, and two were hospitalized due to worsening heart failure. Notably, no patients discontinued bempedoic acid due to adverse effects. Overall, these findings confirm the efficacy of bempedoic acid in improving lipid profiles while maintaining a favorable safety profile in elderly patients with statin intolerance or limited access to PCSK9 inhibitors.

## Discussion

CVDs remain a leading cause of global morbidity and mortality, with dyslipidemia being a major modifiable risk factor. Statins are the cornerstone of lipid-lowering therapy, effectively reducing LDL-C levels by inhibiting HMG-CoA reductase, which leads to upregulation of LDL receptors and enhanced clearance of circulating LDL particles. However, a subset of patients fails to achieve target LDL-C levels despite high-intensity statin therapy, while others experience SI, primarily due to muscle-related adverse effects. Consequently, alternative lipid-lowering strategies have gained increasing attention.

Ezetimibe, a cholesterol absorption inhibitor, reduces LDL-C levels by blocking Niemann-Pick C1-like protein 1 (NPC1L1) at the intestinal brush border, achieving reductions of up to 22% as monotherapy and up to 27% when combined with statins (Cannon et al., 2015).

More recently, PCSK9 inhibitors, such as evolocumab and alirocumab, have revolutionized lipid management by preventing LDL receptor degradation, leading to LDL-C reductions of up to 60% (Gargiulo et al., 2024b; Sabatine et al., 2017; Schwartz et al., 2018). Additionally, Inclisiran, an RNA interference-based therapy, suppresses PCSK9 synthesis, reducing LDL-C levels by approximately 50% with biannual administration (Ray et al., 2020).

BA has emerged as a promising non-statin lipid-lowering agent. It inhibits ATP-citrate lyase (ACLY) to reduce hepatic cholesterol synthesis (Ferri et al., 2024). Clinical trials have demonstrated that BA lowers LDL-C by 18%–28% as monotherapy or in combination with ezetimibe, providing an effective alternative for patients who cannot tolerate high-dose statins (Ray et al., 2019).

This study confirms the efficacy and safety of BA in elderly patients over 81 years at high cardiovascular risk, particularly those who are not eligible for PCSK9 inhibitors due to reimbursement restrictions. Over 52 weeks, BA significantly reduced total cholesterol, LDL-C, and triglycerides while HDL-C levels remained stable. Increased uric acid represented the only significant adverse effect, consistent with BA-induced inhibition of renal OAT2, but remained clinically manageable without treatment discontinuation. Renal and liver function showed no significant changes, supporting the favorable safety profile of BA. Additionally, BA lowers hs-CRP, a key marker of inflammation associated with atherosclerosis (Reddy and Deoker, 2024).

Since chronic low-grade inflammation is crucial in CVD, particularly in elderly patients, BA’s dual lipid-lowering and anti-inflammatory properties may provide an additional cardiovascular benefit. While our study did not assess hs-CRP levels, future investigations should explore the potential of BA in reducing inflammation-mediated cardiovascular risk. From a clinical perspective, BA fills a significant therapeutic gap for elderly patients unable to tolerate statins or access PCSK9 inhibitors. The CLEAR Outcomes trial recently demonstrated a significant reduction in major adverse cardiovascular events (MACE) with BA, further supporting its role in cardiovascular risk management (De Filippo et al., 2023).

However, this study has some limitations. The single-centre design and relatively small cohort size may limit the generalizability of our findings. The lack of a control group also prevents direct comparisons with other lipid-lowering therapies. The low number of cardiovascular events observed during follow-up did not allow for meaningful subgroup comparisons between patients with and without events. Larger, adequately powered studies are needed to explore potential predictors of cardiovascular outcomes in elderly patients treated with bempedoic acid. Larger, multicenter studies with extended follow-up periods are needed to confirm the long-term cardiovascular benefits of BA in elderly populations.

## Conclusion

BA represents a well-tolerated and effective lipid-lowering therapy for elderly patients at high cardiovascular risk, particularly those with statin intolerance or limited access to PCSK9 inhibitors. Our findings support its integration into clinical practice as an alternative treatment option. Further research is warranted to explore its long-term cardiovascular benefits and potential role in inflammation reduction.

## Data Availability

The raw data supporting the conclusions of this article will be made available by the authors, without undue reservation.
